# *GCK* gene mutations are a common cause of childhood‐onset MODY (maturity‐onset diabetes of the young) in Turkey

**DOI:** 10.1111/cen.13121

**Published:** 2016-07-05

**Authors:** Belma Haliloglu, Gerald Hysenaj, Zeynep Atay, Tulay Guran, Saygın Abalı, Serap Turan, Abdullah Bereket, Sian Ellard

**Affiliations:** ^1^ Department of Pediatric EndocrinologyMarmara University Medical SchoolIstanbulTurkey; ^2^ Institute of Biomedical and Clinical ScienceUniversity of Exeter Medical SchoolExeterUK

## Abstract

**Objective:**

Inactivating heterozygous mutations in the *GCK* gene are a common cause of MODY and result in mild fasting hyperglycaemia, which does not require treatment. We aimed to identify the frequency, clinical and molecular features of *GCK* mutations in a Turkish paediatric cohort.

**Design and Patients:**

Fifty‐four unrelated probands were selected based on the following criteria: age of diagnosis ≤17 years, family history of diabetes in at least two generations, anti‐GAD/ICA negative, BMI<95.p and follow‐up with diet, oral antidiabetic drug or low‐dose insulin treatment (≤0·5U/kg/d). A MODY probability score (www.diabetesgenes.org) was calculated and 21 patients with a score ≥75%, HbA1c levels ≤7·5% (58·5 mmol/mol) and fasting blood glucose (FBG) levels 99–145 mg/dl (5·5–8·0 mmol/l) were selected for Sanger sequencing of the *GCK* gene. Targeted next‐generation sequencing for all known monogenic diabetes genes was undertaken for any patient without a *GCK* gene mutation.

**Results:**

*GCK* gene mutations (pathogenic or likely pathogenic variants) and a novel intronic variant of uncertain significance (c.208 + 3A>T) were identified in 13/54 probands (24%). Twelve of these patients had a MODY probability score ≥75%. FBG level and 2‐h glucose level in OGTT were 123 ± 14 mg/dl (6·8 ± 0·7 mmol/l) (107–157 mg/dl) and 181 ± 30 mg/dl (10·1 ± 1·6 mmol/l) (136–247 mg/dl), respectively. Average of glucose increment in OGTT was 58 ± 27 mg/dl (3·2 ± 1·5 mmol/l) (19–120 mg/dl), and mean HbA1c level was 6·5 ± 0·5% (47·5 ± 5·5 mmol/mol) (5·9–7·6%). Five novel missense mutations were identified (p.F123S, p.L58P, p.G246A, p.F419C, and p.S151C). Two patients treated with low‐dose insulin before the molecular analysis were able to stop treatment.

**Conclusions:**

Approximately 1 in 4 MODY cases in this Turkish paediatric cohort have a *GCK* mutation. Selection of patients for *GCK* gene analysis using the MODY probability score was an effective way of identifying most (11/12) patients with a *GCK* mutation.

## Introduction

Glucokinase (GCK) is the enzyme which catalyses the first step of glycolysis and regulates insulin secretion as a glucose sensor.^1^, ^2^ This enzyme is encoded by the *GCK* gene on chromosome 7p15·3‐p15·1 comprising 12 exons and has three tissue‐specific isoforms due to three different‐sized exon 1.^3^ Inactivating heterozygous mutations in the *GCK* gene cause GCK‐MODY (maturity‐onset diabetes of the young) that is characterized by asymptomatic, nonprogressive and mild fasting hyperglycaemia from birth.^2^, ^4^ Glucose increment in oral glucose tolerance test (OGTT) (0–120 min) is less than 54 mg/dl (3 mmol/l) in most cases.^5^ HbA1c levels are just above the normal range and usually between 5·6 and 7·3% (37·7–56·3 mmol/mol) in these patients.^6^ Microvascular and macrovascular complications are rare in patients with *GCK* mutations, and pharmacological treatment is rarely required.^4^, ^7^

To date, there are no publications that investigate the frequency of *GCK* mutations among MODY patients in the Turkish population. In this study, we aimed to determine the frequency of *GCK* mutations in a paediatric Turkish MODY cohort and to identify the molecular and clinical characteristics of GCK‐MODY patients.

## Material and methods

### Patients and selection criteria

**Figure 1:**
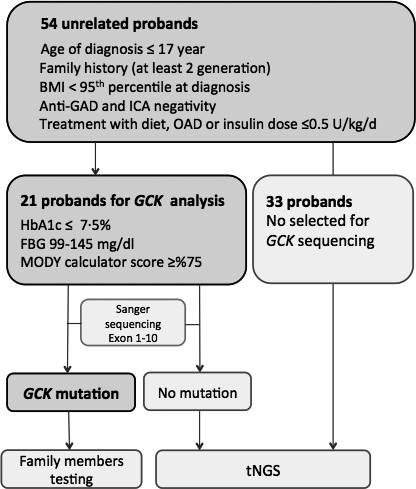
Study schematics indicating steps and selection criteria. OAD *,* Oral antidiabetic drug.

This study analysed 629 children with diabetes that were followed up between 2000 and 2013 at Marmara University Hospital, Department of Pediatric Endocrinology, Istanbul, Turkey. Among them, 61 patients who had a clinical diagnosis of MODY were evaluated. Fifty‐four of these patients (26 males and 28 females) with a mean age at diagnosis of 8·6 ± 4·2 years consented to participate in this study and fulfilled the inclusion criteria, which were the age of diagnosis ≤17 years, a minimum two‐generation family history of diabetes, body mass index (BMI) <95th percentile at diagnosis, negative tests for glutamic acid decarboxylase (GAD) and islet cell (ICA) auto‐antibodies and treatment with diet, oral antidiabetic drug (OAD) or low‐dose insulin treatment (≤0·5 U/kg/d) (Fig. 1). The clinical parameters such as birthweight, BMI, HbA1c, fasting blood glucose (FBG), C‐peptide and/or insulin levels, standard oral glucose tolerance test (OGTT) (2 h) and current treatment were recorded.

The genetic testing strategy is illustrated in Fig. 1. *GCK* sequencing was performed in 21 patients with a FBG level between 99 and 145 mg/dl (5·5–8·0 mmol/l), HbA1c levels ≤7·5% (58·5 mmol/mol) and MODY calculator scores ≥75% as calculated on www.diabetesgenes.org. The description and evaluation of the MODY probability calculator score has been described in detail elsewhere.^8^ All patients without a *GCK* gene mutation underwent targeted next‐generation sequencing (tNGS) analysis as described by Ellard *et al*.^9^in 2013.

### Molecular and data analysis

DNA was extracted for all the probands using the AUTOPURE LS^™^ nucleic acid purification instrument (©QIAGEN, Venlo, Netherlands). After PCR amplification, the minimal promoter region, exons 1–10 and flanking intronic regions of the *GCK* gene (reference sequence NM_000162.3) were Sanger‐sequenced using an ABI 3730 DNA sequencer (©Applied Biosystems, Foster City, CA, USA). Sequence alignment and variant calling were performed using the Mutation Surveyor software (©SoftGenetics, State College, PA, USA).

To determine the pathogenic status, *in silico* predictions were performed using Alamut Visual, Interactive Biosoftware, Rouen, France v2.7.2 software package. A set of different methods were applied, which include SIFT, Align GVGD and PolyPhen‐2 for amino acid changes and SpliceSiteFinder‐like, MaxEntScan, NNSPLICE, GeneSplicer and Human Splicing Finder for splicing predictions. Exome sequencing data generated by the ExAC (Exome Aggregation Consortium) version 0.3 were also accessed. Conservation analyses were performed in the UCSC Genome Browser (http://genome.ucsc.edu/) using the Human GRCh38/hg38 assembly as reference.

Family members of the probands who had a likely pathogenic *GCK* variant were tested to check for cosegregation. Microsatellite analysis was performed on families where the variant was not detected in the proband's parents. Novel variants were classified according to the ACMG guidelines.^10^

Targeted next‐generation sequencing (tNGS) was performed using an exon‐capture assay with baits for 29 genes, as described previously.^9^

The study was approved by the local ethical committee, and the patients or their parents provided informed consent for the study.

## Results

### Molecular genetic test results

A total of 21 probands of 54 unrelated possible MODY cases were selected for *GCK* testing. Eleven different *GCK* pathogenic or likely pathogenic variants were identified in 11/21 patients (52%). One additional *GCK* variant was identified by a targeted next‐generation sequencing assay for all known monogenic diabetes genes in the remaining 43 patients. Each proband was heterozygous for a different variant and these included 10 missense and two splice site variants (Table 1). We also found one novel synonymous exonic variant (p.A173A) with no predicted impact on protein function or mRNA splicing, which was therefore classified as a likely benign variant of no clinical significance. Seven of the variants have been reported previously in the literature (c.46‐2A>G, c.1254‐1G>C, p.K169R, p.R191Q, p.V367M, p.R275C and p.M393T).^3^, ^11^, ^12^, ^13^, ^14^ The remaining five (p.F123S, p.L58P, p.G246A, p.F419C and p.S151C) are not listed in any publications collated by the online Human Gene Mutation Database and were therefore classified as novel (Table 2). In addition, we identified a novel intronic variant, c.208 + 3A>T, of uncertain significance.

**Table 1:** Molecular and clinical characteristics of the patients with *GCK* gene variants

Pt No	Sex	cDNA variants	Amino acid change	Birthweight percentile^a^	Affected parent^e^	Mother treatment in pregnancy	Age at diagnosis	BMI percentile	Maximum HbA1c(%)	Fasting glucose (mg/dl)	2‐h glucose (mg/dl)	Glucose increment in OGTT	Treatment at diagnosis	MODY score	Previously reported
P1	M	c.368T>C	p.F123S	<10.p	Mother	Insulin	1,5	–	7,6	107	198	91	insulin	>75%	Novel
P2^b^	F	c.823C>T	p.R275C	50–90.p	Mother	No	13,3	5–10.p	6,3	127	247	120	insulin	15%	Guazz
P3	M	c.173T>C	p.L58P	10.p	No	–	5,7	25–50.p	7,1	123	152	29	diet	>75%	Novel
P4	M	c.572G>A	p.R191Q	90–97.p	Mother	No	9,3	10–25.p	6,3	116	188	72	diet	>75%	Massa
P5	F	c.46‐2A>G	intronic	> 97.p	Father	No^c^	11,1	10–25.p	6,4	129	185	56	diet	>75%	Estalell
P6^d^	F	c.208 + 3A>T	intronic	50–90.p	Mother	No	0,9	–	6,3	117	136	19	diet	>75%	Novel
P7	F	c.1178T>C	p.M393T	50–90.p	Mother	No	0,4	–	6,3	145	186	41	diet	>75%	Osbak
P8	M	c.737G>C	p.G246A	97.p	Mother	No	9,3	90–95.p	5,9	112	151	39	diet	>75%	Novel
P9	F	c.1256T>G	p.F419C	90–97.p	Mother	No	10,6	75.p	6,7	157	210	53	diet	>75%	Novel
P10	F	c.1254‐1G>C	intronic	90–97.p	Father	–	15,8	10–25.p	6,5	122	180	58	diet	>75%	Osbak
P11	M	c.452C>G	p.S151C	25–50.p	Father	–	5,4	50–75.p	6,5	114	180	66	diet	>75%	Novel
P12	M	c.1099G>A	p.V367M	90–97.p	Mother	No	14,8	5–10.p	6,6	117	191	74	diet	>75%	Velho
P13	M	c.506A>G	p.K169R	50–90.p	Father	–	4,9	50–75.p	5,9	109	148	39	diet	>75%	Ellard^f^

a Adjusted according to gestational week.

b MODY calculator score was >75% for all patients except Patient 2. She was diagnosed via tNGS.

c Mother had GDM without GCK variant.

d P6 has variant of uncertain significance.

e All diabetic and nondiabetic parents were studied, and the variants were detected just in affected parents.

f Ellard S, unpublished data.

**Table 2:** Investigations into the pathogenicity of the novel *GCK* variants identified by this study

Pt ID	Pathogenicity class	Nucleotide description	Protein description	SIFT	PolyPhen‐2	Align GVGD	Amino acid/nucleotide conservation across 11 species^a^	Splicing Prediction	ExAC^b^	Affected family member
P1	Likely Pathogenic	c.368T>C	p.F123S	Likely Pathogenic	Likely Pathogenic	Likely Pathogenic	Conserved in all species	Not predicted to affect splicing	Not listed	Mother
P3	Likely Pathogenic	c.173T>C	p.L58P	Likely Pathogenic	Likely Pathogenic	Likely Pathogenic	Conserved in all species	Not predicted to affect splicing	Not listed	*De novo*
P6	Uncertain significance	c.208 + 3A>T	Intronic	N/A	N/A	N/A	Conserved in all species	Predicted to abolish splice donor site	Not listed	Mother
P8	Likely Pathogenic	c.737G>C	p.G246A	Likely Pathogenic	Likely Benign	Likely Pathogenic	Conserved in all species	Not predicted to affect splicing	Not listed	Mother
P9	Likely Pathogenic	c.1256T>G	p.F419C	Likely Pathogenic	Likely Pathogenic	Likely Pathogenic	Conserved in all species	Not predicted to affect splicing	Not listed	Mother and sibling
P11	Likely Pathogenic	c.452C>G	p.S151C	Likely Pathogenic	Likely Pathogenic	Likely Pathogenic	Conserved in all species	Not predicted to affect splicing	Not listed	Father

a Human, Rhesus, Mouse, Cat, Elephant, Platypus, Chicken, Xenopus Tropicalis, Tetraodon, Zebrafish (UCSC Genome Browser http://genome.ucsc.edu/)

b Not listed in 121412 alleles analysed by the Exome Aggregation Consortium (ExAC), Cambridge, MA (URL: http://exac.broadinstitute.org)

Testing of parental samples showed that the p.L58P variant had arisen *de novo* and is therefore highly likely to be pathogenic. Microsatellite analysis confirmed family relationships. In all other cases, the variants were inherited from a parent with fasting hyperglycaemia characteristic of GCK MODY.

**Figure 2:**
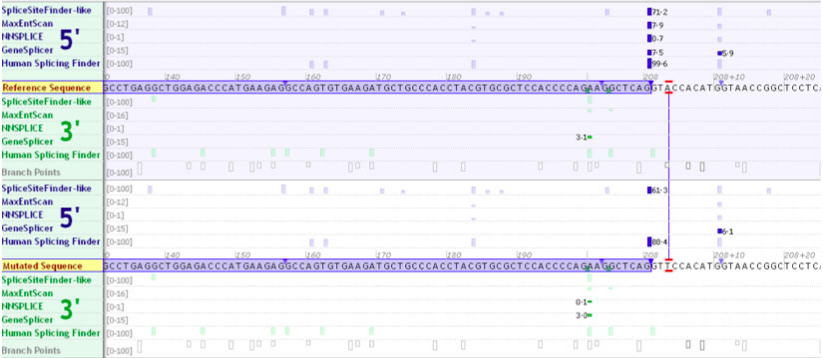
The *In silico* analyses’ result of the novel c.208 + 3A>T splice side mutation. Alamut v2·3 screen view showing the effect of the novel mutation c.208 + 3A>T on the donor splice site of exon 2 of the *GCK* gene. Arrow is indicating the reduced/missing predicted scores for this splice site due to the mutation. Ten nucleotides to the right appears a predicted cryptic donor splice site, which is potentially used instead of the existing splice site.

*In silico* analysis of the novel missense variants predicted that five of the 6 are likely to be pathogenic, with p.G246A classified as uncertain (Table 2). However, reports of other mutations at this codon (p.G246)^3^, ^15^, ^16^ suggest that this residue is important for enzymatic function and therefore this variant is also likely to be pathogenic. Other mutations at codons p.F123, p.S151 and p.F419 have been reported.^3^, ^17^, ^18^, ^19^ The novel splice site variant c.208 + 3A>T was inherited from the proband's affected mother. It is predicted to disrupt the sequence of the donor splice site of exon 2 and promote the use of a neighbouring cryptic donor splice site ten bases downstream (Fig. 2). Use of this splice site would result in the inclusion of an additional ten nucleotides leading to a frameshift and consequently to a premature termination codon in exon 3. As a result, the mRNA is predicted to be degraded by nonsense‐mediated decay. Patient mRNA samples were not available for analysis. Therefore, this variant was classified as of uncertain significance.

### Clinical features of the patients

We identified 12 patients with a *GCK* mutation (pathogenic or likely pathogenic variant) and one variant of uncertain significance. The frequency of GCK‐MODY was 22% (12/54) in the MODY cohort and 1.9% (12/629) in all diabetic children who were followed in our hospital. With the exception of one patient who was diagnosed via targeted next‐generation sequencing, all of the patients’ MODY calculator scores were higher than 75%. The mean age of diagnosis was 7·9 ± 5·2 years (0·4–15·8 years). The mean BMI was 17·2 ± 1·9 kg/m^2^ (14·5–21·2 kg/m^2^), and all patients had normal weight, except for patient 8 (P8) who was overweight. Average birthweight was 3270 ± 860 g (1350–4800 g), but three patients were born prematurely (Table 1).

Mean fasting blood glucose (FBG) level and 2‐h glucose level in OGTT were 123 ± 14 mg/dl (6·8 ± 0·7 mmol/l) (107–157 mg/dl) and 181 ± 30 mg/dl (10·1 ± 1·6 mmol/l) (136–247 mg/dl), respectively. Average 2‐h glucose increment in OGTT was 58 ± 27 mg/dl (3·2 ± 1·5 mmol/l) (19–120 mg/dl), and only 50% (6/12) of the patients had an increment less than 54 mg/dl (3 mmol/l). Mean HbA1c level at presentation was 6·5 ± 0·5% (47·5 ± 5·5 mmol/mol) (5·9–7·6%). Two patients were treated with low‐dose insulin (0·2–0·3 U/kg/d) before the molecular analysis due to high glucose increment in OGTT (91 and 120 mg/dl (5·1 and 6·66 mmol/l), respectively). Patient 1 had the highest HbA1c level (max 7·6% (59·6 mmol/mol)). His diabetic mother was also heterozygous for the same *GCK* mutation and had developed diabetic retinopathy (background retinopathy, <5 microaneurysms) at the age of 32 years. After the genetic diagnosis of GCK MODY was made, insulin treatment was discontinued in both patients. Their mean HbA1c levels were 7·2/7·4% (55·2/57·4 mmol/mol) and 6·2/6·3% (44·3/45·4 mmol/mol) on/off insulin treatment, respectively.

## Discussion

### Molecular characteristics

We detected 12 pathogenic or likely pathogenic *GCK* variants and one intronic variant of uncertain significance. These include 6 previously unreported missense or splicing variants. Two splice site mutations (c.46‐2A>G and c.1254‐1G>C) have already been published as pathogenic.^3^, ^11^ The novel variant c.208 + 3A>G is predicted to affect the donor splice site of exon 2 (Fig. 2). *In silico* analysis predicts that the new donor splice site results in frameshift in the mRNA and causes a premature stop codon, but studies of patient mRNA are required to confirm this. The novel missense variants p.F123S, p.G246A, p.F419C and p.S151C affect residues where other mutations have been reported.^3^, ^15^, ^16^, ^17^, ^18^, ^19^

In 12 probands, the *GCK* variant was inherited from a parent with fasting hyperglycaemia. In the remaining family, the novel p.L58P variant was shown to have arisen *de novo*. Interestingly, a study by Baltrusch *et al*.^20^has investigated the interaction between glucokinase and glucokinase‐regulating protein (GRP). This protein is mainly expressed in the hepatocytes, but its interactions with glucokinase can reveal structural hotspots that can disrupt glucokinase function if mutated. Their findings indicate that the three‐dimensional structure of glucokinase brings the L58 and N204 amino acid residues to close vicinity. Their interaction creates a motif crucial for the interaction with GRP. Although this interaction does not have a direct link to the persistent hyperglycaemia observed in our patient, it indicates that the L58 amino acid residue is very important in the three‐dimensional folding of glucokinase. In addition, the N204 residue is part of the glucose‐binding site of glucokinase. Because of the close proximity between L58 and N204 in the three‐dimensional structure of glucokinase, a mutation in the L58 may perturb the conformation at the active site and affect the glucose binding to glucokinase.^20^

### Clinical characteristics

A correct diagnostic approach and early diagnosis of GCK‐MODY is important to avoid unnecessary investigations and pharmacological treatment. In this cohort of 54 Turkish paediatric cases, 11 *GCK* mutations were identified in 21 suspected GCK‐MODY children (52%) and one *GCK* mutation in the remainder of the cohort.

The frequency of GCK‐MODY varies according to different healthcare systems between countries which affect ascertainment. Whereas its frequency in children with MODY is 83% in Poland, it is 22·5%, 41% and 41% in Japan, Spain and Italy, respectively.^12^, ^21^, ^22^, ^23^ Approximately 1 in 4 MODY cases (24%) in this cohort are due to *GCK* mutations. A recent study showed a minimum population prevalence of 1 in 1000 in the Atlantic‐DIP study.^24^

Genetic testing is the gold standard for diagnosis of GCK‐MODY and is cost‐effective in clinically selected patients.^25^ Selection techniques such as the MODY probability calculator can assist with the identification of those patients most likely to have a *GCK* mutation.^8^ In this study, *GCK* analysis was performed only for the patients with fasting blood glucose between 99 and 145 mg/dl (5·5–8·0 mmol/l), HbA1c levels ≤7·5% (58·5 mmol/mol) and a MODY calculator score ≥75%. This strategy proved effective as a high frequency of *GCK* mutations was detected in this group (52%) but only one *GCK* mutation in the rest of the cohort. This unusual patient had a low MODY probability calculator score (15%) due to insulin treatment at diagnosis. The other patient who was currently on insulin treatment did not start insulin treatment until 1 year later after diagnosis, so the MODY calculator score was >75%.

The glucose increment in OGTT is less than 54 mg/dl (3 mmol/l) in 70%^5^ and 83 mg/dl (4·6 mmol/l) in 95% of patients with GCK‐MODY (Ellard and Hattersley, unpublished data). Only two mutations have been reported that cause exceptionally high postprandial glucose levels.^26^ Additionally, HbA1c levels are lower in GCK‐MODY than other types of MODY and diabetes.^4^ In a recent study, HbA1c reference range in GCK‐MODY children was 5·6–7·3% (37·7–56·3 mmol/mol) and provided good sensitivity and specificity for discriminating hyperglycaemia likely to be caused by a *GCK* mutation.^6^ Although the HbA1c levels in this study were within expected range except one, the glucose increments in OGTT were slightly higher than expected.

The patients with GCK‐MODY have similar incidence of micro‐ or macrovascular complications compared with controls without diabetes.^7^ Steele *et al*. reported that only diabetic retinopathy frequency (30%) was higher than controls, but all were background retinopathy that is not clinically significant.^7^ None of our patients with GCK‐MODY in our cohort had any micro‐ or macrovascular complication, but the mother of P1 developed diabetic retinopathy that did not require laser treatment.

Pharmacological treatment is not recommended in GCK‐MODY.^4^, ^27^ Stride *et al*. found that GCK–MODY patients are unresponsive to pharmacological therapy as a consequence of their glucose sensing defect.^27^ In our cohort, only two patients were treated with insulin at the time of genetic testing. The pharmacological treatment was discontinued after the molecular analyses and HbA1c levels did not rise when insulin treatment was stopped.

This study provides first data about the frequency and molecular–clinical features of GCK‐MODY in a Turkish paediatric population. It also demonstrates the utility of using clinical characteristics in combination with a MODY probability score to select patients with a very high likelihood of GCK‐MODY.

## Funding

Belma Haliloglu has received a grant from the International Society for Pediatric and Adolescent Diabetes (ISPAD) for this work. The present study was also supported by the Turkish Society for Pediatric Endocrinology and Diabetes.

## Conflict of interest statement

The authors report there are no conflicts of interest.
